# Helical Blade Versus Lag Screw Fixation in Cephalomedullary Nailing of Unstable Trochanteric Fractures: A Narrative Review

**DOI:** 10.7759/cureus.114278

**Published:** 2026-08-10

**Authors:** Muhammad Haris Irfan, Henilkumar R Patel

**Affiliations:** 1 Norwich Medical School, University of East Anglia, Norwich, GBR; 2 Postgraduate Department, Manchester University NHS Foundation Trust, Manchester, GBR; 3 Department of General Surgery, Royal Bolton Hospital, Bolton, GBR

**Keywords:** cephalomedullary nail, dynamic hip screw, helical blade, hip fracture fixation, intertrochanteric fracture, lag screw, tip-apex distance, trochanteric fracture

## Abstract

Trochanteric (intertrochanteric) hip fractures are among the most common fragility fractures in older adults. Cephalomedullary nailing (CMN) has become the standard treatment strategy for unstable fracture patterns (AO/OTA 31-A2/A3), largely replacing the sliding hip screw (dynamic hip screw, DHS) for this indication. Two proximal fixation designs are in widespread use within CMN systems: the traditional lag screw, which relies on thread engagement in cancellous bone, and the helical blade, which is inserted by impaction, compacting surrounding cancellous bone rather than removing it. This narrative review synthesises the clinical, biomechanical, registry-based, and health-economic evidence comparing helical blade and lag screw fixation. This review also discusses how these findings relate to the wider evidence on implant selection, technical factors influencing fixation success, and complications following CMN, including peri-implant fracture, nonunion, and salvage arthroplasty. Across systematic reviews, meta-analyses, cadaveric biomechanical studies, and large registry cohorts, no consistent superiority of either proximal fixation device has been demonstrated with respect to overall fixation failure, including cut-out. Failure patterns nonetheless differ between implant designs. Medial perforation occurs exclusively with helical blades in the largest single-centre series to report this distinction, while superior migration is more commonly observed with lag screws. Helical blade performance may additionally vary with patient frailty and bone quality. Health-economic evidence continues to favour extramedullary fixation for stable fracture patterns and CMN for unstable fractures. A tip-apex distance greater than 25 mm remains the most reproducible predictor of fixation failure regardless of implant type, while reduction quality and implant positioning are consistently more important determinants of outcome than proximal fixation device selection. Implant choice should therefore be individualised according to patient frailty, bone quality, and surgeon familiarity.

## Introduction and background

Trochanteric (intertrochanteric) fractures occur at the proximal femur, distal to the femoral neck. Cephalomedullary nailing (CMN) is a widely used surgical technique in which an intramedullary nail is inserted along the femoral shaft, with a head-fixation device passing through the nail into the femoral neck and head to secure the fracture during healing; this head-fixation device may take the form of a lag screw, a threaded implant that achieves purchase through thread engagement in cancellous bone, or a helical blade, a non-threaded implant inserted by impaction. The tip-apex distance (TAD), referenced throughout this review, quantifies the distance from the tip of the head-fixation device to the apex of the femoral head on radiographs and is among the most established predictors of fixation failure ("cut-out"). Trochanteric fractures account for roughly half of all hip fractures and carry substantial morbidity and mortality in the elderly population [1]. Ninety-day mortality is modestly higher after intertrochanteric fracture than after femoral neck fracture (12.1% vs. 9.6% in one large series), with advanced age, comorbidity burden, and delayed surgery identified as key risk factors [2]. Contemporary management guidelines accordingly emphasise orthogeriatric co-management, individualised treatment based on biological rather than chronological age, and early mobilisation [3]. Surgical fixation options broadly divide into extramedullary devices, principally the DHS, and intramedullary cephalomedullary nails. For stable two-part fracture patterns (AO/OTA 31-A1), the two implants generally perform comparably [4]. For unstable patterns (31-A2/A3), however, CMN is now favoured. Biomechanical testing has shown superior load-sharing and lower peak cancellous bone stresses with CMN when fractures are anatomically reduced [4]. Clinically, a meta-analysis found lower revision rates than DHS augmented with a trochanteric stabilisation plate, although complication rates and functional outcomes were similar [5]. 

Despite the widespread adoption of CMN for unstable trochanteric fractures, uncertainty remains regarding the optimal proximal fixation design. Whether a helical blade or a traditional lag screw reduces the risk of mechanical cut-out remains debated [6-9]. Related questions include whether single-screw nail systems (PFNA and Gamma3) differ from dual-screw or integrated designs such as InterTAN [10,11]. This review synthesises the current evidence comparing helical blade and lag screw fixation, places these findings within the wider evidence on CMN design, and discusses the influence of technical factors, health-economic considerations, and complications beyond cut-out. 

## Review

Methods

This is a narrative review rather than a systematic review. Relevant literature was identified through an iterative, topic-focused search of PubMed/MEDLINE for English-language articles published between January 2014 and July 2026, using combinations of the terms "cephalomedullary nail," "helical blade," "lag screw," "intertrochanteric fracture," "trochanteric fracture," "tip-apex distance," "dynamic hip screw," "cut-out," "PFNA," "InterTAN," "Gamma nail," and "cost-effectiveness," supplemented by hand-searching the reference lists of retrieved articles for additional relevant studies. As this was an iterative, topic-focused search rather than a single reproducible database query, exact numbers of records identified, screened, and excluded were not formally tracked.

We considered randomised controlled trials, prospective and retrospective comparative cohort studies, systematic reviews, meta-analyses, cadaveric biomechanical studies, finite element analyses, and health-economic decision-analysis studies for inclusion. Case reports were included selectively where they illustrated a mechanism of failure relevant to the discussion, such as the limits of cement augmentation. Reference lists of included reviews were hand-searched to identify additional relevant studies. We excluded studies restricted to paediatric populations, pathologic fractures, and periprosthetic fractures around pre-existing implants, except where the latter arose as a fixation-failure complication. As this was a narrative review rather than a systematic review, formal risk-of-bias assessment and PRISMA-style study selection were not performed. Nonetheless, two authors (MHI and HRP) independently screened titles, abstracts, and full texts against the inclusion and exclusion criteria described above; disagreements regarding study eligibility were resolved by discussion and consensus between the two authors. Findings are synthesised qualitatively in the sections that follow, beginning with the biomechanical rationale for comparing helical blade and lag screw fixation.

Biomechanical rationale

The two proximal fixation designs achieve purchase in the femoral head by fundamentally different mechanisms. The lag screw is inserted along a pre-drilled and tapped path and relies on thread engagement with cancellous bone for axial and rotational stability. The helical blade, by contrast, is impacted along its helical geometry without prior reaming, compacting the surrounding cancellous bone rather than removing it, a property theorised to be advantageous in osteoporotic bone where thread purchase is otherwise poor [12].

**Figure 1:**
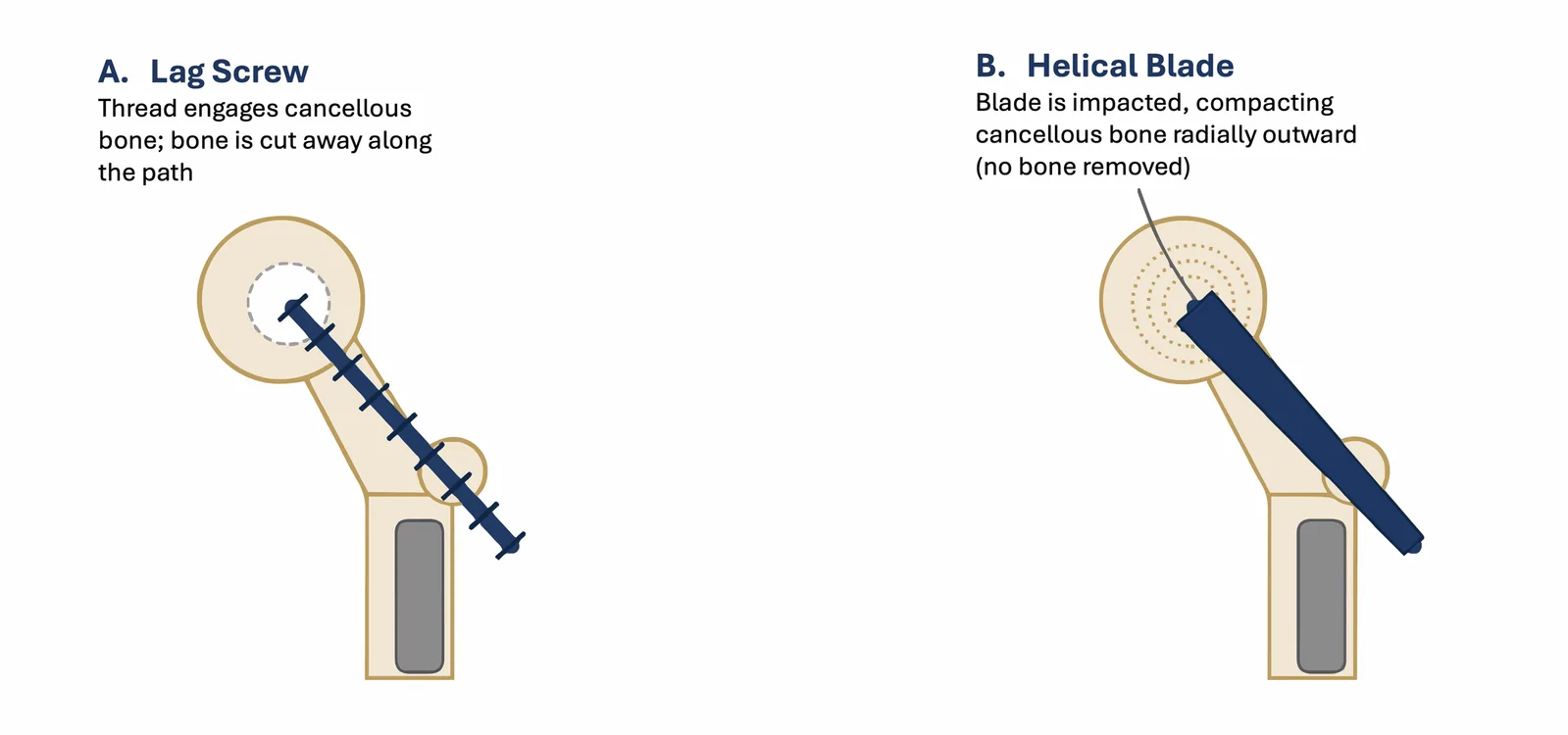
Schematic comparison of the mechanisms by which the lag screw and helical blade achieve fixation in cancellous bone The lag screw (A) relies on thread purchase in cancellous bone, which is removed along the insertion path; the helical blade (B) is press-fit and compacts surrounding cancellous bone rather than removing it, a property theorised to improve fixation in osteoporotic bone. Image credit: The figure was created by the authors using Microsoft PowerPoint.

Cadaveric biomechanical studies provide partial support for this compaction rationale. In a paired human cadaveric model of osteoporotic and osteopenic bone, Pastor et al. found that helical blade fixation (TFN-ADVANCED) performed comparably to an interlocking dual-screw design (InterTAN) in osteopenic specimens, and that cement augmentation of the helical blade significantly increased stiffness and fixation strength relative to interlocking screws in osteoporotic bone [12]. Finite element and sawbone-based testing by Kyriakopoulos et al. showed that, in unstable fracture patterns reduced anatomically, CMN constructs produced lower peak cancellous bone stresses than DHS constructs. Varus malreduction increased implant and bone stress substantially regardless of implant type, suggesting that reduction quality, rather than implant selection, is the dominant determinant of mechanical outcome [4]. This pattern is also reflected in the clinical literature. Figure 1 illustrates these contrasting fixation mechanisms schematically: the lag screw achieves purchase through thread engagement in cancellous bone, whereas the helical blade is impacted and compacts the surrounding bone rather than removing it.

Clinical evidence: helical blade versus lag screw

Despite a plausible biomechanical rationale favouring the helical blade in osteoporotic bone, clinical studies have not consistently demonstrated superiority over lag screw fixation. The largest study to date, a retrospective cohort of 22,308 CMNs from an integrated US health system, found no significant difference in 10-year aseptic revision risk between helical blade and lag screw fixation overall (adjusted HR 0.87, 95% CI 0.69-1.11) [6]. The helical blade did outperform the lag screw in healthier patients (ASA 1-2: adjusted HR 0.65, 95% CI 0.43-0.98), but this advantage disappeared among patients with an ASA classification of 3 or higher [6]. These findings suggest that patient frailty and comorbidity burden, rather than implant type alone, modify relative device performance.

A systematic review and meta-analysis by Ng et al. reached a similar conclusion, pooling 12 studies (745 lag screws, 371 helical blades) and finding no significant difference in cut-out rate between the two fixation types (OR 1.03, 95% CI 0.25-4.23) [7]. The same analysis identified a TAD greater than 25 mm as the dominant modifiable risk factor for cut-out (OR 3.72, 95% CI 2.06-6.72), a point we return to below. Single-centre retrospective series have produced more heterogeneous findings. Ibrahim et al. reviewed 313 patients (245 helical blades, 68 lag screws) and found no overall difference in cut-out rate but identified a mechanistic divergence: all cases of medial perforation involved helical blades, while superior migration occurred significantly more often with lag screws. These findings suggest that the two implants may fail by different mechanisms despite similar overall failure rates [8]. Chong et al., incorporating DEXA T-scores and alkaline phosphatase as bone-quality markers, found similar one-year complication and survivorship rates between the two implants regardless of bone quality, but a higher five-year implant survivorship for helical blades in osteoporotic patients (77% vs. 69%), a finding that fits the compaction rationale but does not prove it [9].

**Figure 2:**
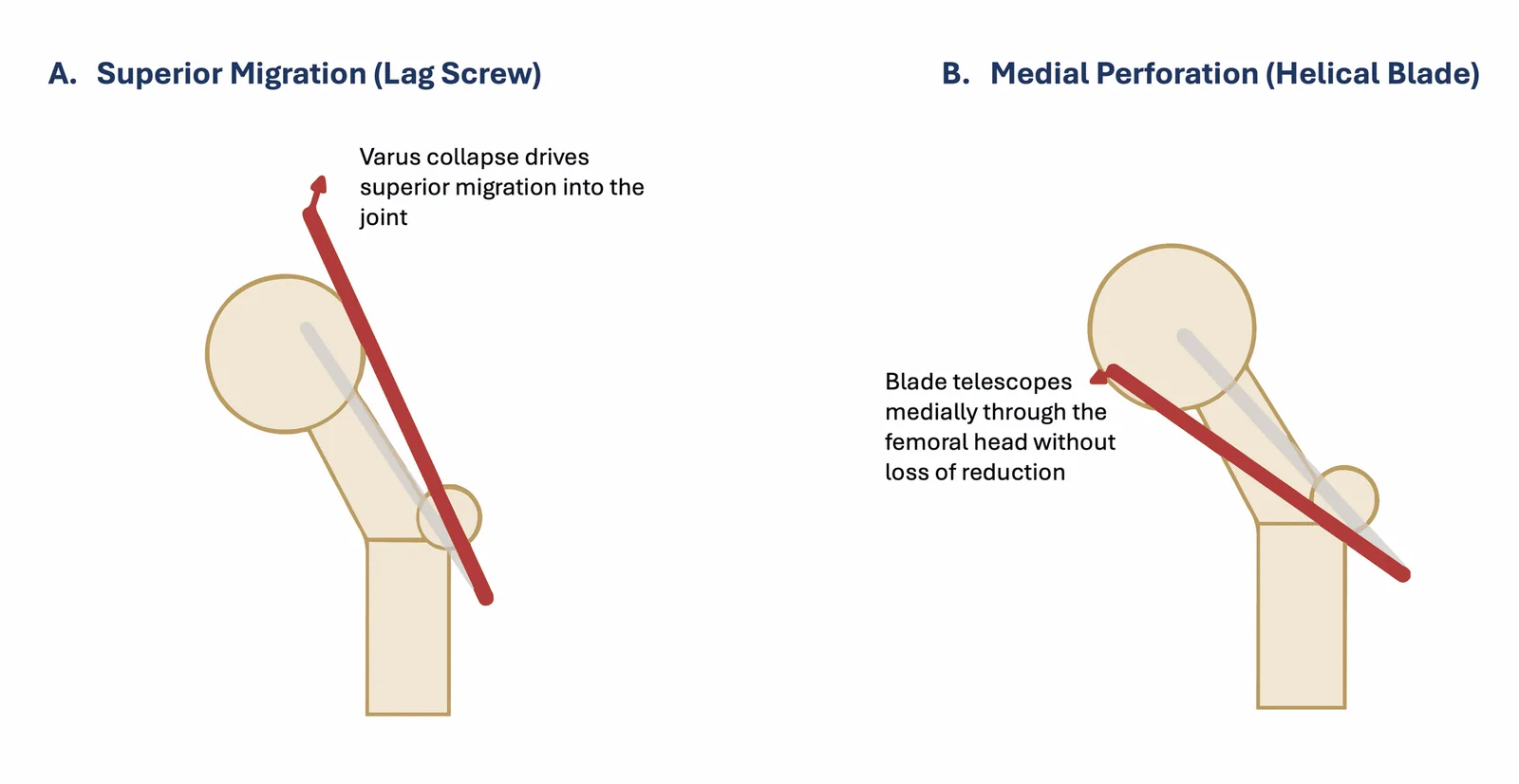
Schematic comparison of the two principal mechanisms of cephalomedullary nail failure Even when overall failure rates are similar between implants, the two devices tend to fail by different mechanisms: lag screws more commonly fail through superior migration accompanying varus collapse (A), whereas helical blades more commonly fail through medial perforation of the femoral head without loss of reduction (B). Image credit: The figure was created by the authors using Microsoft PowerPoint.

Surgical technique modifications applicable to either device also appear to influence outcomes independent of implant choice. Goodnough et al. found that countersinking the lag screw or blade past the lateral cortex reduced implant prominence and fracture collapse without increasing cut-out risk [13]. Karapınar et al. showed that inferior-posterior helical blade placement was as safe as central placement, which expands the acceptable positioning envelope for blade fixation [14]. Whether the head element is a blade or a screw is, however, only one axis of nail design; how many screws engage the femoral head at all is another. Figure 2 illustrates the two principal failure mechanisms discussed above: superior migration accompanying varus collapse with lag screw fixation and medial perforation of the femoral head with helical blade fixation.

Nail system design: single-screw versus dual-screw constructs

A related but distinct design question concerns whether integrated dual-screw or dual-element systems, such as InterTAN, which uses interlocking compression screws, outperform single lag screw or single helical blade systems such as PFNA and Gamma3. A 2025 meta-analysis of 24 comparative studies (2,999 patients) found broadly similar functional recovery across the three devices. PFNA offered shorter operative times but had higher cut-out and revision rates; InterTAN, despite longer operative times, was associated with faster union and fewer complications. Gamma3 did not outperform either alternative on any measured outcome [10].

An earlier meta-analysis by Ma et al. similarly found InterTAN associated with a lower cut-out rate and fewer femoral fractures compared with single-screw systems, with no significant differences in Harris Hip Score, blood loss, union time, hospital stay, or revision rate [11]. Despite this cut-out signal, Ma et al. nevertheless concluded that the available evidence did not support recommending InterTAN as the preferred alternative intramedullary nail, reasoning that the mechanical advantage did not translate into superior functional recovery or revision-free survival and came at the cost of longer operative time [11]. The two meta-analyses, published eight years apart, reach different practical conclusions from broadly similar data: the earlier one advised against routine InterTAN adoption despite its cut-out advantage, while the more recent one favoured InterTAN for complex fractures. These differing conclusions illustrate that a lower cut-out rate alone has not consistently been considered sufficient to justify changing implant selection when other outcomes are equivalent and operative time is longer. A Cochrane systematic review of randomised and quasi-randomised trials across multiple nail-design comparisons, including PFN versus Gamma nail and PFNA versus Gamma3, similarly found the trial evidence insufficient to establish meaningful differences between designs [15]. An independent meta-analysis restricted to integrated dual-screw versus single-screw constructs found lower rates of all-cause revision, cut-out, and screw migration with the dual-screw design, but no corresponding advantage in Harris Hip Score or other functional measures [16]. Regardless of implant design, however, cut-out is most consistently predicted by implant positioning, particularly TAD, which is discussed below.

The tip-apex distance rule in the cephalomedullary era

The most widely studied positional measurement is the TAD. The TAD was originally described for extramedullary sliding hip screws, and its applicability to cephalomedullary constructs, which achieve fixation by a different biomechanical mechanism, was initially uncertain. John et al. tested this directly in 75 patients with unstable trochanteric fractures treated with CMN and found that TAD remained a significant predictor of cut-out (mean TAD 28.8 mm in cut-out cases versus 19.4 mm in those without, p = 0.002), with a calculated threshold of approximately 23.6 mm, closely mirroring the classic 25 mm threshold described for DHS [17]. Suboptimal device positioning and poor restoration of neck-shaft angle were independently associated with cut-out on univariate analysis, though not on multivariate analysis, suggesting these factors interact rather than act independently [17]. Kim et al., in a series of 353 patients treated with three different CMN systems, similarly found a lower mean TAD in patients without complications, though TAD did not reach significance as an independent risk factor on multivariate analysis once reduction quality was accounted for [18].

Refinements to the TAD concept continue to emerge. Çepni et al. proposed a tip-neck distance ratio that favours infero-posterior screw placement and found it to be an independent predictor of mechanical complications alongside reduction quality [19]. A standardised TAD normalised to the patient's own femoral head diameter has similarly been proposed and found to be an independent predictor of cut-out on multivariate analysis, with no cut-outs observed below a threshold of roughly one-third of femoral head diameter [20]. A separate retrospective analysis of 158 patients found that TAD referenced to the calcar (calTAD) discriminated cut-out risk more strongly than conventional TAD, with values above 24.95 mm associated with a markedly increased risk of cut-out [21]. Computer-assisted surgical techniques may also improve the accuracy of implant positioning. Kuhl and Beimel found that computer-assisted lag screw placement reduced median TAD (13 mm vs. 16 mm) and markedly lowered the proportion of cases exceeding the 25 mm threshold (1% vs. 12%), although the study was underpowered to detect a corresponding reduction in cut-out rate [22].

Complications beyond cut-out

Although cut-out is the most frequently reported complication following CMN, several other complications are relevant when comparing fixation strategies.

Peri-implant and periprosthetic fracture

Peri-implant fracture is an uncommon but recognised complication of intramedullary fixation. Halonen et al., reviewing 987 consecutive PFNA fixations, reported an overall peri-implant fracture rate of 1.4%, with no statistically significant difference between short, intermediate, and long nail constructs, arguing against routine use of longer nails purely to mitigate this risk [23].

Nonunion and salvage arthroplasty

Kim et al. found nonunion to be an uncommon but recognised complication following CMN (3 of 353 cases), distinctly less common than cut-out (26 cases) in the same series [18]. When CMN fixation fails, particularly via lag screw cut-out, conversion to total hip arthroplasty (CTHA) is a recognised salvage pathway, but Schultz et al. found that CTHA after failed CMN carries substantially higher morbidity than elective primary arthroplasty, with increased operative time, blood loss, length of stay, and readmission rates, and residual leg-length discrepancy averaging 6.7 mm even after successful conversion [24]. These findings highlight that the consequences of cut-out extend well beyond index reoperation.

Limits of cement augmentation

Cement augmentation of the helical blade has been proposed to mitigate cut-out risk in severely osteoporotic bone, and cadaveric data support a biomechanical benefit [12]. However, a recent case report by Yokoo et al. highlights that augmentation does not eliminate the need for accurate implant positioning: despite TAD and calTAD values within accepted targets, a cement-augmented helical blade construct progressed to subcapital femoral neck fracture over 15 months, attributed to a cleavage plane forming along the inferior cement mantle once sliding capacity was exhausted [25]. Although limited to a single case, this report reinforces that cement augmentation should be regarded as an adjunct to, rather than a substitute for, accurate implant positioning and preserved telescoping capacity. Beyond their clinical consequences, these complications also have important economic implications, providing the rationale for cost-effectiveness considerations that follow.

Cost-effectiveness considerations

The blade-versus-screw and CMN-versus-DHS debates carry economic as well as mechanical implications. Swart et al. constructed a decision-analysis model comparing sliding hip screw and intramedullary nail fixation across AO/OTA fracture subtypes and found that the sliding hip screw remained more cost-effective for stable (A1) fractures and, in 70% of modelled scenarios, for fractures of questionable stability (A2), while intramedullary nailing dominated (lower cost and better outcomes) for reverse-obliquity (A3) fractures; results were highly sensitive to the assumed fixation failure rate [26]. At the level of proximal fixation-device selection within CMN systems specifically, the cost-effectiveness model by Nherera et al. found that an integrated twin compression screw nail was associated with cost savings of $1,652-$3,280 per patient compared with single lag screw or single helical blade nails, driven primarily by fewer modelled complications, though this analysis relied on a pairwise meta-analysis of heterogeneous comparative studies rather than head-to-head randomised data [27]. Overall, the cost-effectiveness literature suggests that fracture stability, rather than helical blade versus lag screw selection per se, is the principal economic determinant of implant choice. Dual-screw or twin-compression designs may offer additional cost advantages through reduced complication rates rather than through the blade versus screw distinction itself.

Summary of comparative evidence

Table 1 summarises the design, comparison, and principal findings of the key studies discussed above. The results column reports each study’s principal statistical findings together with a brief explanation of what those values indicate. A level of evidence grading is provided alongside each study to help readers gauge the strength of the underlying evidence.

**Table 1:** Summary of key comparative studies CAS: computer-assisted surgery; CMN: cephalomedullary nailing; DHS: dynamic hip screw; HR: hazard ratio (values below 1.0 favour the first-listed device; values above 1.0 favour the comparator); IMN: intramedullary nail; OR: odds ratio (values below 1.0 favour the first-listed device; values above 1.0 favour the comparator; a 95% confidence interval including 1.0 indicates the difference is not statistically significant); SHS: sliding hip screw; TAD: tip-apex distance; TSP: trochanteric stabilisation plate; p-values below 0.05 were considered statistically significant. LOE: level of evidence, graded according to the widely used Levels of Evidence hierarchy for Primary Research Question originally developed by the Journal of Bone and Joint Surgery (JBJS) (therapeutic studies, Levels I-V, based on study design) and for Economic and Decision Analyses (Levels I-V, based on data quality and use of sensitivity analysis); levels are as self-reported by study authors where explicitly stated in the original publication, and otherwise assigned by the review authors based on study design. Therapeutic and economic-analysis LOE scales are not directly comparable; the study by Swart et al. is graded on the economic-analysis scale, while all other studies in this table are graded on the therapeutic scale.

Study	Design (n)	Comparison	Results	Key Finding
Okike et al., 2025 [6] (LOE III)	Registry cohort, n=22,308	Blade vs screw	10-year aseptic revision (overall): 1.69% (blade) vs 1.88% (screw); adjusted HR 0.87 (95% CI 0.69–1.11), not statistically significant. 10-year aseptic revision (ASA I– II, healthier patients): adjusted HR 0.65 (95% CI 0.43–0.98), favouring blade. 10-year aseptic revision (ASA III or higher, frailer patients): adjusted HR 1.03 (95% CI 0.78–1.35), no difference.	Blade fixation was associated with lower revision risk only in healthier patients (ASA I-II); no advantage was observed in ASA III or higher.
Ng et al., 2021 [7] (LOE III)	Meta-analysis, 12 studies, n=1,116	Blade vs screw	Cut-out: OR 1.03 (95% CI 0.25–4.23), not statistically significant. TAD >25 mm as a risk factor for cut-out: OR 3.72 (95% CI 2.06–6.72), significantly increased odds.	TAD >25 mm was the strongest predictor of cut-out (OR 3.72, i.e., nearly four-fold higher odds)
Ibrahim et al., 2019 [8] (LOE III)	Retrospective cohort, n=313	Blade vs screw	Cut-out rate: 15/245 blade (6.1%) vs 5/68 screw (7.4%); p=0.45, not statistically significant. Failure mechanism: medial perforation occurred exclusively with blades; superior migration occurred more often with screws.	Distinct failure modes: all medial perforations occurred with blades; superior migration occurred more often with screws
Chong et al., 2023 [9] (LOE III)	Retrospective cohort, n=303	Blade vs screw, by bone quality	1-year complication rate: 16% (blade) vs 22% (screw), not statistically significant. 5year implant survivorship (osteoporotic bone): 77% (blade) vs 69% (screw), favouring the helical blade	Five-year implant survivorship favoured the helical blade in osteoporotic bone (77% vs 69%)
John et al., 2019 [17] (LOE III)	Retrospective cohort, n=75	TAD as predictor	Mean TAD: 28.8 mm (cut-out cases) vs 19.4 mm (no cut-out); p=0.002, statistically significant. Estimated cut-out threshold: ~23.6 mm	TAD rule remains valid for CMN, with an estimated cut-out threshold of ~23.6 mm
Kuhl & Beimel, 2020 [22] (LOE III)	Retrospective comparative, n=204	CAS vs conventional placement	Proportion with TAD >25 mm: 1% (computer-assisted) vs 12% (conventional). Cut-out rate difference between groups: not statistically significant (study underpowered to detect this outcome).	Computer-assisted surgery reduced TAD variability but was underpowered to detect a difference in cut-out rate.
Selim et al., 2021 [5] (LOE III)	Meta-analysis, five studies	CMN vs DHS + TSP	Revision rate: lower with CMN than DHS + TSP (pooled meta-analysis; effect size not reported in original studies). Complication rate and functional outcome: no significant difference between constructs.	CMN was associated with a lower revision rate, with no significant difference in complication rate or functional outcome.
Miller et al., 2025 [10] (LOE III)	Meta-analysis, 24 studies, n=2,999	PFNA vs Gamma3 vs InterTAN	Complication rate: lowest with InterTAN. Operative time: shortest with PFNA. Cut-out and revision rate: highest with PFNA. Gamma3: did not lead on any measured outcome	No single device was consistently superior across all measured outcomes; device choice may reasonably depend on fracture complexity.
Ma et al., 2017 [11] (LOE II)	Meta-analysis (RCTs/cohorts)	InterTAN vs Gamma/PFNA	Cut-out rate and femoral fracture rate: lower with InterTAN. Harris Hip Score, blood loss, union time, hospital stay, and revision rate: no significant difference between devices	Despite lower cut-out and femoral fracture rates with InterTAN, the authors did not recommend its routine adoption because functional outcomes, revision rates, and most other outcomes were similar, while operative time was longer.
Swart et al., 2014 [26] (LOE II, economic analysis)	Decision-analysis model	SHS vs IMN (cost)	Cost-effectiveness (stable A1 and borderline-stable A2 fractures): SHS more cost-effective. Cost-effectiveness (reverse-obliquity A3 fractures): IMN dominant (lower cost and better outcomes). Sensitivity analysis: results highly sensitive to the assumed implant failure rate.	SHS was more cost-effective for A1 and most A2 fractures, whereas IMN was favoured for A3 fractures; these conclusions were highly sensitive to the assumed implant failure rate.

**Figure 3:**
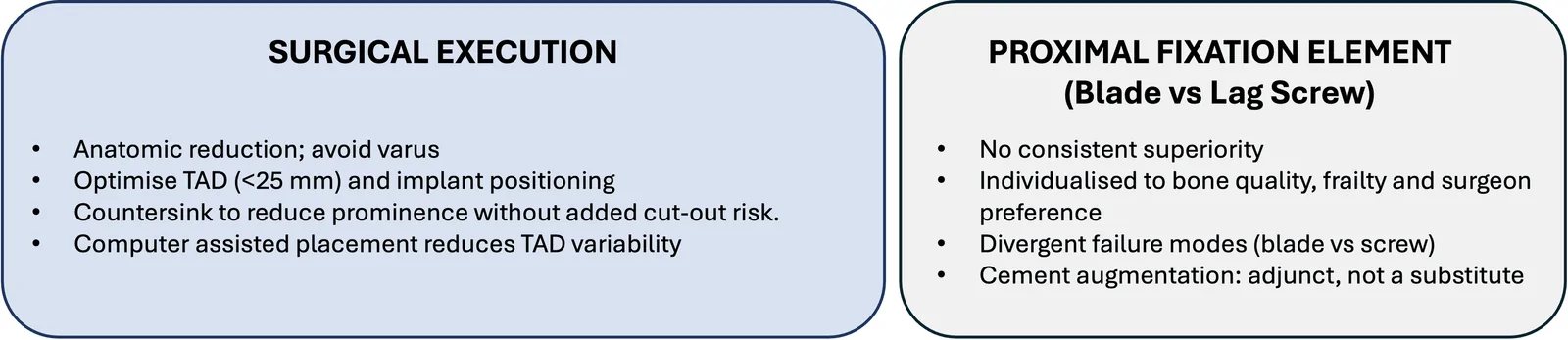
Conceptual framework for fixation success in cephalomedullary nailing of trochanteric fractures. Conceptual summary of the principal factors influencing fixation success following cephalomedullary nailing for trochanteric fractures. Reduction quality, implant positioning, and optimisation of the tip-apex distance (TAD) are more consistently associated with fixation success than the choice between a helical blade and a lag screw. Image credit: The figure was created by the authors using Microsoft PowerPoint.

Table 1 summarises the comparative evidence in tabular form, whereas Figure 3 synthesises the same evidence into a conceptual framework, highlighting that fixation success depends primarily on surgical execution and appropriate device selection rather than implant choice alone. 

Discussion

Proximal Fixation Element: Blade, Lag Screw, and Single-Screw Versus Dual-Screw Design

The current evidence does not support a categorical mechanical advantage for either the helical blade or the lag screw in CMN of unstable trochanteric fractures. The largest registry data [6] and the most comprehensive available meta-analysis [7] both point toward overall equivalence in cut-out and revision risk. Where differences do emerge, they are patient-specific (a possible advantage for helical blades in healthier, lower-ASA patients) or mechanistic (differing failure modes: medial perforation with blades, superior migration with screws) rather than a uniform superiority of one device. In practice, this argues for individualising implant selection to patient frailty, bone quality, and surgeon familiarity rather than applying a fixed rule. 

The single-screw versus dual-screw comparison offers a somewhat clearer, though still partial, signal. Dual-screw and integrated twin-compression designs (InterTAN, ITCS) are associated with lower cut-out and femoral fracture rates than single-screw systems across two independent meta-analyses separated by nearly a decade [10,11], and a cost-effectiveness model attributes savings to this reduced complication burden [27]. This signal is not unanimous in its clinical interpretation; however, the earlier of the two meta-analyses explicitly declined to recommend routine InterTAN adoption because the cut-out advantage did not translate into better functional recovery, revision-free survival, or operative efficiency [11], while the more recent analysis was more favourable, particularly for complex fracture patterns [10]. These findings raise the possibility that rotational control at the femoral head, rather than the compaction versus threading mechanism of the head element itself, may be the more important design variable. Whether a lower cut-out rate alone should drive implant selection, in the absence of a matching functional or revision-rate benefit, remains a matter of genuine disagreement among authors rather than a settled question. 

Surgical Execution

What the literature does support consistently is that surgical execution outweighs implant selection. TAD greater than 25 mm remains among the most reproducible predictors of cut-out across both DHS and CMN systems and across both lag screw and helical blade designs [7,17], although at least one large single-centre series found TAD lost independent significance once reduction quality was included in multivariate modelling [18]. Reduction quality, device positioning within the femoral head, and avoidance of varus malalignment are consistently identified as stronger determinants of mechanical failure than the choice between a helical blade and a lag screw [4,8]. Adjunct techniques such as countersinking, computer-assisted placement, and attention to infero-posterior quadrant positioning may offer more reliable gains in fixation reliability than the choice of proximal fixation device itself [13,14,22]. Cement augmentation may extend the margin for error in severely osteoporotic bone but does not substitute for accurate positioning, as illustrated by reported late failures despite augmentation [25]. Finally, when fixation does fail, the consequences are clinically significant: conversion arthroplasty after failed CMN carries meaningfully higher morbidity than elective primary arthroplasty [24], reinforcing that failure-prevention strategies (reduction quality, TAD, positioning) have downstream value beyond the index procedure. These conclusions, however, rest on an evidence base with its own constraints, which we outline below. 

Limitations

This narrative has several limitations. As a narrative rather than systematic review, study selection was not exhaustive, no formal risk-of-bias assessment or PRISMA flow diagram was applied, and publication bias favouring positive or novel findings cannot be excluded. The evidence base itself is dominated by retrospective, single-centre cohorts with variable follow-up, heterogeneous fracture classification, and inconsistent reporting of bone quality and reduction grade, which limits direct comparison across studies. Randomised comparisons of helical blade versus lag screw specifically, as opposed to broader nail-system comparisons, remain scarce, and the largest dataset available [6], while providing unmatched statistical power, remains a retrospective registry-based comparison and is therefore susceptible to residual confounding despite propensity weighting. Cost-effectiveness estimates are model-based and highly sensitive to assumed failure rates and regional cost inputs, limiting generalisability across health systems. This heterogeneity extends beyond fracture classification to encompass surgeon and institutional learning curves: several of the included studies, particularly those evaluating computer-assisted placement and quadrant-based blade positioning, report outcomes from surgeons or centres with variable prior experience with the technique under study, and the reported benefits of these approaches may not generalise to less experienced practitioners or lower-volume centres. Follow-up duration also varies considerably across the included studies, from short-term radiographic outcomes at a few months to implant survivorship reported at five or 10 years; shorter follow-up may underestimate late complications such as delayed cut-out or peri-implant fracture, while direct comparison of studies with differing follow-up windows should be made cautiously. The literature search was also restricted to a single database (PubMed/MEDLINE); although reference lists of included reviews were hand-searched to identify additional studies, this approach may still have omitted relevant literature indexed only in other databases such as Embase, Scopus, or the Cochrane Library, and this database selection bias should be considered when interpreting the completeness of the evidence synthesised here. Table 1 additionally presents a meta-analysis addressing the blade-versus-screw question directly (Ng et al., pooling 12 studies) alongside some of the individually listed primary studies on the same comparison (e.g., Ibrahim et al., Chong et al.); potential overlap between the primary studies contributing to this pooled analysis and the individually listed studies was not systematically verified, and readers should not interpret the number of table entries as reflecting an equivalent number of fully independent patient cohorts. With these caveats in mind, the following conclusions represent our best synthesis of the current evidence. 

## Conclusions

In CMN of unstable (AO/OTA 31-A2/A3) trochanteric fractures, helical blade and lag screw fixation demonstrate broadly similar rates of mechanical cut-out and revision, though they may fail by different mechanisms and may perform differently across patient frailty strata. Dual-screw and integrated twin-compression nail designs show more consistent complication-rate advantages than the blade-versus-screw distinction alone. Implant selection should be individualised according to bone quality, fracture pattern, and patient comorbidity, and further prospective, adequately powered comparative data, ideally randomised, are needed to resolve the blade-versus-screw question definitively.

The TAD rule, originally developed for extramedullary devices, remains a broadly applicable predictor of fixation failure in cephalomedullary nailing, although its independent significance may be attenuated when reduction quality is included in multivariable models. Current evidence nonetheless suggests that meticulous surgical technique, reduction quality, implant positioning, and optimisation of the TAD have greater influence on fixation success than the choice between a helical blade and a lag screw. Surgeons should recognise that fixation failure carries meaningful downstream morbidity via salvage arthroplasty, underscoring the clinical importance of these technical factors regardless of the implant selected.
